# The prevalence of autoimmune hepatitis is rising: Estimates and trends from a large, multi-ethnic cohort in the United States

**DOI:** 10.1097/HC9.0000000000000824

**Published:** 2025-10-07

**Authors:** Jimmy Yao, Sheng-Fang Jiang, Chandni Kapoor, Sripriya Balasubramanian, Nirmala D. Ramalingam, Varun Saxena, Kavita Radhakrishnan

**Affiliations:** 1 Department of Medicine, Kaiser Permanente Oakland Medical Center, Oakland, California, USA; 2 Division of Research, Kaiser Permanente Northern California, Pleasanton, California, USA; 3 Department of Gastroenterology, The Permanente Medical Group, Kaiser Permanente Roseville, South San Francisco, California, USA; 4 Department of Graduate Medical Education, Kaiser Permanente Oakland Medical Center, Oakland, California, USA; 5 Department of Gastroenterology and Transplant Hepatology, The Permanente Medical Group, Kaiser Permanente South San Francisco Medical Center, South San Francisco, California, USA; 6 Department of Gastroenterology and Transplant Hepatology, University of California, San Francisco, California, USA; 7 Department of Gastroenterology, The Permanente Medical Group, Kaiser Permanente Oakland Medical Center, Oakland, California, USA

**Keywords:** age, demographics, epidemiology, integrated healthcare, race

## Abstract

**Background:**

Large epidemiologic studies of autoimmune hepatitis (AIH) in the United States are limited. None have reported prevalence trends over time. This contemporary study examines AIH prevalence and demographic trends over 10 years in a community-based integrated healthcare system in Northern California. We further assessed whether prevalence trends differed by AIH ascertainment approach.

**Methods:**

This retrospective study used data from adults aged ≥18 years in Kaiser Permanente Northern California (2010–2019). AIH was identified by coded diagnosis and confirmed with diagnostic testing (laboratory and/or liver biopsy) and treatment response. Annual AIH prevalence was estimated and stratified by age, sex, and race/ethnicity.

**Results:**

Among 1129 patients with confirmed AIH, 80% were female, 44% non-Hispanic White, 26% Hispanic, 16% Asian/Pacific Islander, and 9% Black. In all, 76% of patients on AIH treatment demonstrated treatment response at 6 months. AIH prevalence (per 100,000 adults) increased from 9.1 in 2010 to 18.8 in 2019 (*p*<0.0001). Prevalence among older adults (≥75 years) quadrupled from 10.1 to 43.7 per 100,000. Prevalence rose among all ethnicities and in 2019 was highest for Black (28.9) and Hispanic populations (25.2) per 100,000.

**Conclusions:**

AIH prevalence doubled over 10 years in a large healthcare system, with pronounced increases among older populations. Prevalence was highest among Black and Hispanic adults. Further studies should examine demographic differences in the clinical course of AIH, including response to therapy, adverse events, and outcomes.

## INTRODUCTION

Autoimmune hepatitis (AIH) is a chronic inflammatory liver disease that can lead to cirrhosis, liver failure, the need for liver transplantation, and death. As with other autoimmune conditions, such as type 1 diabetes, psoriasis, and inflammatory bowel disease,^1^^,^^2^ AIH prevalence is rising globally.^3^ Accurate estimates of prevalence trends in AIH in the United States are not readily available due to the absence of comprehensive national patient registries or databases. Consequently, there is a paucity of population-based studies pertaining to AIH, with no contemporary studies evaluating prevalence trends in AIH over time.

Two recent studies^4^^,^^5^ that evaluated the epidemiology of AIH in the United States at a national level using administrative data and International Classification of Diseases (ICD) codes for AIH reported point prevalence estimates of ~26.5–31.2 per 100,000 persons. These studies demonstrated markedly elevated prevalence in the elderly, mirroring global studies.^6^ Both studies utilized different data sets and algorithms to identify AIH cases. Specifically, Tunio et al.^4^ utilized the Explorys aggregate Health Record and identified AIH patients by ICD code but excluded those with an additional diagnosis of viral hepatitis, alcohol-associated liver disease, and drug-induced liver disease. Bittermann et al.^5^ utilized the Optum Clinformatics Data Mart and identified AIH by ICD code and excluded those with primary biliary cholangitis (PBC), primary sclerosing cholangitis (PSC), and/or immunotherapy prescription. Notably, these studies had differing racial and ethnic prevalence estimates, with Bittermann et al.^5^ demonstrating the highest AIH prevalence in Black and Hispanic persons, while Tunio et al.^4^ demonstrated high prevalence among Asian and lower prevalence among Black populations, with differences between studies hypothesized to be due to varying methodologies of identification of AIH cases and/or source population differences.^5^

More studies, including studies conducted among diverse populations with similar access to healthcare, are necessary to build upon this existing literature and to more clearly understand AIH prevalence and demographic differences in clinical settings. However, few US studies to date have examined AIH prevalence trends within a single healthcare system, where laboratory, biopsy findings, and treatment response could be used to support accurate ascertainment of AIH.

This study examined demographic characteristics of adults with AIH and trends in prevalence by age, sex, and race/ethnicity over a 10-year period in a diverse, representative, community-based US healthcare population. Using data from an integrated healthcare delivery system, comprehensive identification of AIH cases allowed assessment of AIH prevalence and trends by method of AIH ascertainment.

## METHODS

### Data source

Electronic health record (EHR) data were used to identify adults aged ≥18 years who received a diagnosis of AIH from 2010 to 2019 at Kaiser Permanente Northern California (KPNC), a large integrated healthcare system with over 4.5 million members. The study was approved by the KPNC Institutional Review Board with waiver of informed consent.

KPNC provides integrated healthcare to 14 counties of the greater Bay Area as well as the California Central Valley and includes patients in both urban and rural areas. This population is highly representative of the demographics of the entire geographic area including race/ethnicity as well as neighborhood-linked income, education and social vulnerability.^7^ All care is provided within a closed system and documented in the electronic health record.

### Case identification and confirmation

Patients with a diagnosis of AIH were initially identified from International Classification of Diseases (ICD) 9th/10th Revision Clinical Modification codes (ICD-9-CM 571.42, ICD-10-CM K75.4). AIH was then confirmed by liver biopsy records when available and/or at least one of the following laboratory criteria: positive antinuclear antibody (ANA), elevated actin immunoglobulin G (≥20 Units), and/or total IgG (≥1600 mg/dL) within 6 months before or after the first AIH-coded diagnosis. Liver biopsy reports were reviewed by clinical and transplant hepatologists, and pathology findings deemed to be likely or possible AIH were included using the recent consensus recommendations from the International AIH Pathology Group.^8^ Those with an AIH diagnosis and positive laboratory findings but inconsistent findings on liver biopsy (eg, steatotic liver disease) were not considered confirmed AIH. Confirmed AIH cases were therefore defined as those with an ICD code for AIH and consistent diagnostic data (biopsy and/or lab).

#### Simplified AIH score

Among confirmed AIH cases, the Simplified diagnostic AIH score was calculated^9^ as a standardized measure of diagnosis verification. This 8-point scoring system was developed for use in clinical practice and utilizes diagnostic laboratory information and liver biopsy results to determine the likelihood of AIH and to help guide the decision to start treatment. A score of ≥6 points is consistent with probable AIH, and a score of 7–8 points is indicative of definite AIH.^9^ To account for those with only partial available data for review, we report an “at least” (minimum) simplified AIH score.

#### Treatment and response to treatment

To further validate this AIH cohort, we next assessed AIH treatment and response to treatment. To have a standardized approach to treatment response, we identified those with a first diagnosis of AIH (incident cases) and evaluated treatment/response in this subset of patients. We looked at standard AIH treatments, including steroid induction and commonly utilized first and second-line therapies.^10^ We divided treatment into steroid (prescription for prednisone and/or budesonide), maintenance (prescription for azathioprine, mercaptopurine, mycophenolate, and/or tacrolimus), or combination steroid + maintenance therapy. Treatment response was defined as normalization of ALT 6 months after diagnosis.

### Statistical analysis

Prevalent diagnoses, examined annually, were defined as confirmed AIH diagnoses captured within a specific year. The annual prevalence of confirmed AIH was examined overall and by age, sex, and race/ethnicity subgroup. The Cochran-Armitage test was used to examine trends in the prevalence of diagnosed AIH. We examined prevalence estimates and trends using 3 AIH cohort definitions:

*AIH cohort I (primary)*: Patients with an ICD-coded diagnosis of AIH are confirmed with any available diagnostic data (any labs and/or confirmatory biopsy data). We then performed sensitivity analyses to determine prevalence estimates for the more selected subset of patients with both an ICD-coded diagnosis of AIH and a confirmatory liver biopsy consistent with AIH (*cohort II*) and the broader population of patients with an ICD-coded diagnosis of AIH, regardless of the availability of diagnostic data or biopsy results (*cohort III*).

## RESULTS

### Identification and confirmation of AIH

**FIGURE 1:**
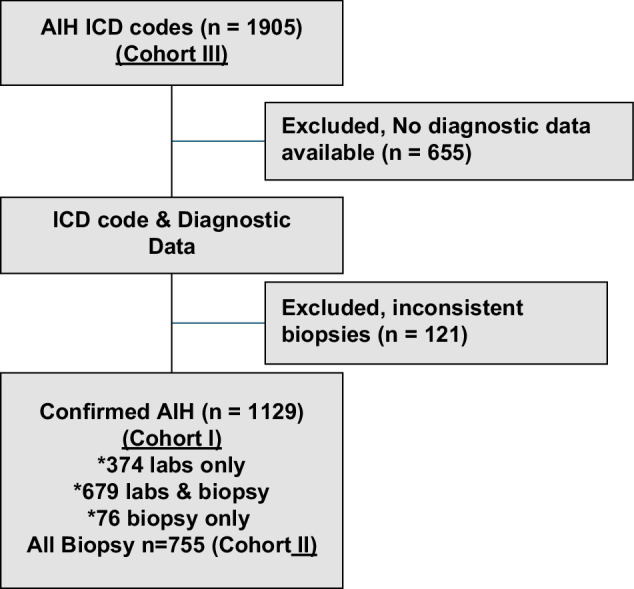
Cohort selection diagram. Abbreviations: AIH, autoimmune hepatitis; ICD, International Classification of Diseases.

There were 1905 adults with potential AIH identified by ICD diagnostic codes for AIH, comprising cohort III. Of these, 655 (34%) had no diagnostic data available for review. Of the remaining 1250 patients, 121 (10%) had a liver biopsy, which revealed an alternative non-AIH diagnosis. A total of 1129 patients, therefore, remained as confirmed AIH (cohort I) with 679 (60%) patients having labs and biopsy diagnostic data available, and an additional 76 (7%) with biopsy data only (comprising 755, cohort II) and 374 (33%) with laboratory data only (Figure 1).

### Liver biopsies

There were 885 patients who had biopsies available for review. In all, 121 patients (14%) were excluded due to liver biopsy confirming an alternative diagnosis, the majority comprising steatotic liver disease (n=35, 29%) and cholestatic/biliary process (n=33, 27%). Twenty patients (17%) had either normal or minimal injury on biopsy, which in the pathology report was felt to be inconsistent with AIH. Another 27% (n=33) had an alternative diagnosis documented by a pathologist in the context of their reported history, including mass, nodular regenerative hyperplasia, drug-induced liver injury, and viral hepatitis. Finally, 9 patients had no available biopsy report or insufficient tissue on liver biopsy (but had labs consistent with AIH and were therefore included in cohort I). Overall, 755 patients had biopsies consistent with AIH (cohort II).

### Characteristics of the confirmed AIH cohort

Of the 1129 patients with confirmed AIH, 898 (80%) were female and 502 (44%) were non-Hispanic White (NHW), 288 (26%) Hispanic, 176 (16%) Asian/Pacific Islander (A/PI), 104 (9%) Black, and 59 (5%) other/unknown race/ethnicity. Half (50%) of all patients with confirmed AIH had an AIH diagnosis between the ages of 55 and 74 during the study period. The baseline demographics of cohorts I, II, and III are shown in Table 1.

**TABLE 1:** Baseline demographics of AIH by cohorts

	Cohort I Confirmatory lab and/or biopsy	Cohort II Confirmatory liver biopsy	Cohort III ICD-coded AIH
	N=1129	N=755	N=1905
Female, n (%)	898 (80)	607 (80)	1492 (78)
Age group^a^, n (%)			
18–34 years	142 (13)	90 (12)	237 (12)
35–54 years	307 (27)	199 (26)	539 (28)
55–74 years	561 (50)	394 (52)	924 (49)
≥75 years	119 (10)	72 (10)	205 (11)
Race/ethnicity, n (%)			
Asian/Pacific Islander	176 (16)	122 (16)	285 (15)
Black	104 (9)	79 (11)	177 (9)
Hispanic	288 (26)	191 (25)	485 (24)
Other/Unknown	59 (5)	41 (5)	133 (7)
Non-Hispanic White	502 (44)	322 (43)	852 (45)

^a^ At first diagnosis during the study period.

Abbreviations: AIH, autoimmune hepatitis; ICD, International Classification of Diseases.

Among AIH cases in cohort I, 13% were aged 18–34 years, 27% aged 35–54 years, 50% aged 55–74 years, and 10% aged ≥75 years at the first ascertained AIH diagnosis during the study period. Two-thirds (n=755, 67%) had liver biopsies consistent with AIH, 936 (83%) had elevated actin IgG levels, 472 (42%) had elevated IgG levels, and 493 (44%) had positive ANA.

### Simplified AIH score

The simplified AIH score was applied to all 1129 patients with confirmed AIH based on any diagnostic data available (cohort I). Incomplete data were predominantly due to a lack of ANA titers, and 40% had no IgG levels, resulting in an “at least” simplified AIH score, which is reported. Despite missing data, two-thirds of patients with liver biopsy (n=506) had a simplified AIH score of ≥6, and 66% of patients without a biopsy (n=245) had a simplified AIH score of ≥4. The range of scores was 2–8. The distribution of scores for cohorts I and II is provided in Supplemental Table S1, http://links.lww.com/HC9/C136.

### Treatment and treatment response

Of the 1129 patients with confirmed AIH, 807 (71%) were incident cases. Within this group, 92 (11%) were on no treatment for AIH. Of the 715 patients on any AIH treatment, 547 (76%) demonstrated treatment response, defined as normalization of ALT at 6 months. The breakdown of treatment (steroid, maintenance, combination) and treatment response is shown in Table 2. The median starting dose of prednisone was 40 mg and budesonide 9 mg.

**TABLE 2:** Treatment and treatment response among 807 new cases of AIH

	Treatment (n, %)	Treatment response ALT normalization at 6 months (proportion, %)
Steroid monotherapy	145 (18.0)	123/145 (84.8%)
Maintenance monotherapy	20 (2.5)	16/20 (80%)
Steroid and maintenance therapy	550 (68.1)	408/550 (74.2%)

Abbreviation: AIH, autoimmune hepatitis.

### Trends in AIH prevalence from 2010 to 2019

#### Cohort I—Confirmed AIH with ICD code and confirmatory diagnostic data (n=1129 cases)

**FIGURE 2:**
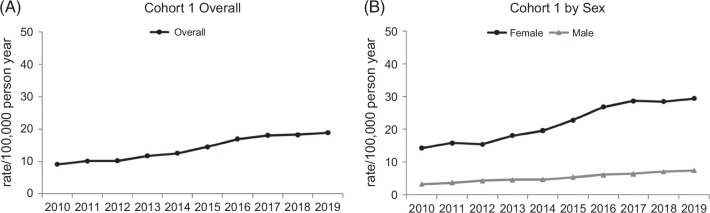
Annual prevalence of AIH per 100,000 individuals: (A) overall versus (B) by sex.

**FIGURE 3:**
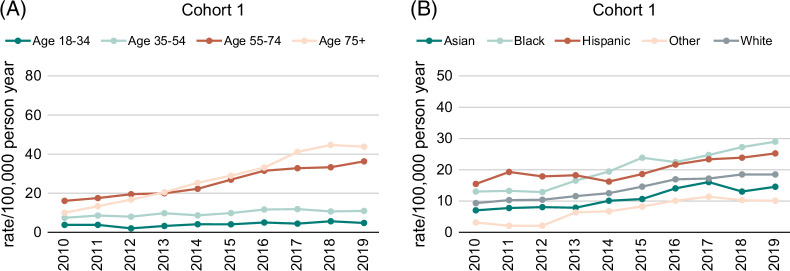
Annual prevalence of AIH per 100,000 individuals by (A) age group and (B) race/ethnicity.

From 2010 to 2019, the prevalence of confirmed AIH (per 100,000 adults) increased from 9.1 in 2010 to 18.8 in 2019 (*p*<0.0001, Figure 2A). Middle-aged (55–74 years) and older adults (≥75 years) had the highest prevalence of diagnosed AIH each year (36.4 and 43.7, respectively) in 2019. Among older adults, the prevalence of diagnosed AIH quadrupled from 10.1 in 2010 to 43.7 in 2019 per 100,000 (*p*<0.0001, Figure 3A). Significant increases were seen among all races and ethnicities examined (Figure 3B). In 2019, the prevalence of AIH per 100,000 adults was 28.9 for Black, 25.2 for Hispanic, 18.5 for White, and 14.5 for Asian persons (Figure 3B). Prevalence rates per 100,000 from 2010 to 2019 doubled for women (14.3–29.4) and men (3.3–7.4), *p*<0.0001 (Figure 2B). The annual distribution of AIH diagnoses among cohort I is shown by sex, age group, and race/ethnicity in Table 3.

**TABLE 3:** Annual distribution of AIH diagnoses among adults, cohort 1

	2010	2011	2012	2013	2014	2015	2016	2017	2018	2019
N	219	250	258	301	340	419	513	571	602	640
Female, %	83	83	80	81	82	82	83	83	81	81
Age group, %										
18–34 years	11	10	6	8	9	8	8	7	9	7
35–54 years	31	32	29	30	24	24	24	24	21	20
55–74 years	49	48	53	48	51	53	53	52	52	55
75+ years	9	10	12	14	16	15	15	17	18	19
Race/Ethnicity, %										
Asian/Pacific Islander	12	12	13	11	14	13	15	17	14	15
Black	10	9	8	10	11	11	9	9	10	10
Hispanic	26	29	28	26	22	23	24	25	25	26
Other/Unknown	5	3	3	6	6	6	6	6	6	6
Non-Hispanic White	47	47	48	47	47	47	46	43	45	43

When restricting AIH cases to the 755 with liver biopsies compatible with AIH (cohort II), prevalence estimates per 100,000 adults increased from 5.2 in 2010 to 14.1 in 2019 (*p*<0.0001, Supplemental Figure S1A, http://links.lww.com/HC9/C134). When including all 1905 patients with AIH ICD codes (cohort III), prevalence estimates per 100,000 increased from 21.7 in 2010 to 28.8 in 2019 (*p*<0.0001, Supplemental Figure S1C, http://links.lww.com/HC9/C134). More detailed trends in prevalence estimates for these 2 cohorts are presented in Supplemental Figure S1, http://links.lww.com/HC9/C134, and S2, http://links.lww.com/HC9/C135.

## DISCUSSION

Our study is the first to examine AIH prevalence and trends from 2010 to 2019 in a single, large integrated healthcare delivery system with comprehensive access to laboratory, pathology, and treatment data. Importantly, we observed that the prevalence of diagnosed AIH doubled from 2010 to 2019, primarily due to an increase in prevalence among older adults. In addition, we examined 3 definitions of AIH to demonstrate the impact of case definition methodology on prevalence estimates and trends. Regardless of whether AIH was broadly defined (including those with an ICD diagnosis, with or without confirmatory data) or more strictly defined (limited to those with confirmatory liver biopsy results, the majority with high simplified AIH score), prevalence is rising overall and particularly among older adults and is most prevalent in Black and Hispanic persons in the United States.

### Autoimmune hepatitis identification

We utilized 3 approaches for identifying AIH patients—namely those with any diagnostic data, those with liver biopsy, and those with an ICD-coded diagnosis. Notably, when looking at prevalence identified by ICD coding alone, the overall prevalence of 28.8 per 100,000 in 2019 was comparable to prior US estimates.^4^^,^^5^ When limiting AIH to those with confirmatory biopsy and/or lab diagnostic data, the prevalence was lower at 18.8 per 100,000 in 2019 and even lower at 14.1 per 100,000 when limiting to the subset with confirmatory biopsy. Furthermore, we found that roughly 14% of those with liver biopsy had an inaccurate ICD-based diagnosis, where liver biopsy review predominantly reflected steatotic liver disease and/or cholestatic processes; this number may be an underestimation given that 34% of patients with an ICD code had no diagnostic data, including biopsy available. While others have suggested algorithms utilizing ICD coding and excluding those with PBC/PSC and script for immunotherapy^11^ or concomitant ICD coding for alternative chronic liver diseases^5^ as options to exclude those with alternative diagnoses, these approaches still include those with steatotic liver disease, which in our cohort was found in a third of excluded cases, and are therefore overestimating AIH prevalence.

While we sought to verify our AIH ascertainment methodology by applying the simplified AIH score to the entire cohort, we were limited by incomplete data. This theme of incomplete data is also seen in other recent studies looking at national AIH trends. For example, the large hallmark study of AIH incidence and prevalence in Denmark which utilized a national registry and was linked to a National Pathology Registry did not have biopsy data on a quarter of patients.^6^ The ICD-based algorithm used to identify AIH patients by Bitterman et al.^4^ was developed within a population of 250 patients, of whom 57% had sufficient records for validation.^11^ The Tunio et al. cohort^5^ had liver biopsies in only 33% of patients. In our AIH ascertainment approaches, cohort II—which included all those with confirmatory liver biopsy— comprised only 40% of those initially identified by AIH ICD coding and was therefore felt to be overly restrictive. However, requiring some form of confirmation of AIH diagnosis beyond ICD coding increases confidence in the accuracy of the cohort. We therefore opted for a “middle-ground” approach by requiring the availability of any form of diagnostic data—laboratory and/or biopsy—as a means of confirming the diagnosis with the goal of more accurately capturing AIH prevalence estimates and trends. We further validated this cohort by exploring treatment and response, noting that of the incident AIH cases, the majority (89%) were on some form of AIH treatment and of those, 76% demonstrated treatment response.

### Prevalence trends over time

With the use of our preferred methodology for identification of cases (cohort I), the overall prevalence of AIH doubled from 2010 to 2019, from 9.1 to 18.8 per 100,000 persons. While trends in prevalence over time were not assessed in the prior US studies, this is in line with global registry-based studies. Incidence rates in Denmark nearly doubled from 1994 to 2014^6^ and from 1997 to 2015 in the United Kingdom.^12^ Prevalence rose from 10.7 to 17.3/100,000 in Sweden from 1990–2003 to 2009.^13^^,^^14^ As these prior studies have noted, there have been no major changes in AIH diagnostic criteria or methodologies that can explain this rise. We additionally looked at the total number of liver biopsies done within KPNC over this time frame and noted they remained consistent annually, providing further support that prevalence is on the rise as opposed to increasing attempts to diagnose AIH.

### Race and ethnicity

The estimation of prevalence trends in different races/ethnicities is a major strength of this paper, as such data are globally understudied. This diverse KPNC AIH cohort is particularly unique, given that patients have similar access to healthcare. Notably, half of all AIH cases occurred in patients from minority groups: 26% Hispanic, 16% A/PI, and 9% Black. As a basis of comparison, the source KPNC population from which this AIH cohort was ascertained is largely similar: 44% NHW, 24% Hispanic, 23% A/PI, 7% Black,^7^ except for a slightly increased proportion of A/PI persons. From 2010 to 2019, we noted a rising prevalence of AIH in all racial and ethnic groups. Our multi-ethnic cohort is reflective of the US population and differs from the US cohorts used previously for estimation of AIH prevalence in that both prior studies included predominantly White populations, with only 1% Hispanic AIH patients in the Tunio study^5^ or 10% in the Optum source population utilized in the Bitterman study.^4^ The latter also utilized primarily imputed race data. Our race/ethnicity trends are in line with the Bitterman et al. findings,^4^ demonstrating the highest prevalence in Black, followed by Hispanic, White, and Asian persons. The fact that this prevalence trend holds with more stringent criteria, namely the availability of confirmatory liver biopsy, strengthens the credibility of our findings. This is highly clinically relevant given that very small prior studies have demonstrated higher rates of cirrhosis in Black and Hispanic AIH patients^15^^,^^16^ as well as increased hospitalizations and higher odds of death.^17^ We plan to look within our diverse population to confirm these findings with the aim of identifying modifiable risk factors.

### Age

Older adults are recognized as an important group with rising AIH prevalence, and our findings are concordant with prior United States and international studies. AIH prevalence rose most notably among older adults aged ≥75 years. Specifically, prevalence quadrupled from 10.1 in 2010 to 43.7 in 2019 per 100,000 (*p*<0.0001), compared with more modest increases in younger adults (age 35–54) from 7.4 in 2010 to 11.0 per 100,000 (*p*<0.0001). This is in line with prior international studies, including a global meta-analysis demonstrating the highest prevalence among adults over age 65 years^3^ as well as country-specific studies from Denmark with peak incidence at age 70 years^6^ and New Zealand, where 45% of patients presented at ages 50–70 years.^18^ These results are also comparable to the prior US studies where peak prevalence was noted at 68.6/100,000 age 75–79^5^ and 72/100,000 ages 70–79.^4^ The cause of this dramatic rise in prevalence requires further investigation, including whether the increase is driven by greater incidence among older patients or improved disease control and longer lifespan of those diagnosed at a younger age. Several studies demonstrate favorable treatment response among older patients^19^^–^^23^ with fewer flares than in younger patients.^24^ Updated contemporary studies characterizing the natural course of AIH, response to therapy, and adverse treatment effects are needed to inform treatment recommendations for this growing population.

### Limitations

Our study has limitations to consider. First, we utilize data from a single integrated healthcare delivery system, which may impact the generalizability of our findings. However, the large size and similar access to care for all patients allow for a unique examination of epidemiologic trends not available using national databases. Second, due to incomplete data, we do not have biopsy and laboratory data for all patients. Lastly, we captured prevalent diagnoses (number of confirmed AIH diagnoses per year); this method may slightly underestimate prevalence if a patient did not have annual visits.

### CONCLUSIONS

In conclusion, we examined AIH trends in a diverse population in a large, integrated healthcare system in Northern California, the majority of whom have confirmatory liver biopsy results. We have demonstrated a rising prevalence overall from 2010 to 2019, driven by rising prevalence in older patients. We found that roughly 1 in 8 patients with an ICD code of AIH and liver biopsy were inaccurately coded. When utilizing more rigorous methods to identify AIH patients, prevalence estimates decrease, but racial/ethnic and age prevalence trends hold, further validating these conclusions. Future studies should examine the potential factors contributing to increasing AIH prevalence and whether there are demographic differences in AIH presentation and outcomes that might inform strategies for optimizing clinical management.

## Supplementary Material
