# Supplementary material for: The prevalence of autoimmune hepatitis is rising: Estimates and trends from a large, multi-ethnic cohort in the United States
Source: Hepatol Commun. 2025 Oct 7;9(11):e0824. doi: 10.1097/HC9.0000000000000824 (PMC12506987; doi:10.1097/HC9.0000000000000824)
Supplement: Supplementary file 1 [file hc9-9-e0824-s001.docx]

**Article Title:** Prevalence Estimates and Trends in Autoimmune Hepatitis in a Large, Multi-Ethnic Cohort in the United States
**First Author:** Jimmy Yao, MD

Supplemental Figure 1a-d, annual AIH prevalence per 100,000 individuals: a. Prevalence in Cohort 2 (confirmed by liver biopsy) overall increased from 5.2 to 14.1 from 2010-2019; b. Prevalence by sex in Cohort 2 for women increased from 8.3 to 22.1 and for men from 1.7 to 5.4 from 2010-2019; c. Prevalence in Cohort 3 (AIH by ICD code) overall increased from 21.7 to 28.8 from 2010-2019; d. Prevalence by sex in Cohort 3 for women increased from 33.6 to 44.3 and for men from 8.1 to 12.1 from 2010-2019.

| 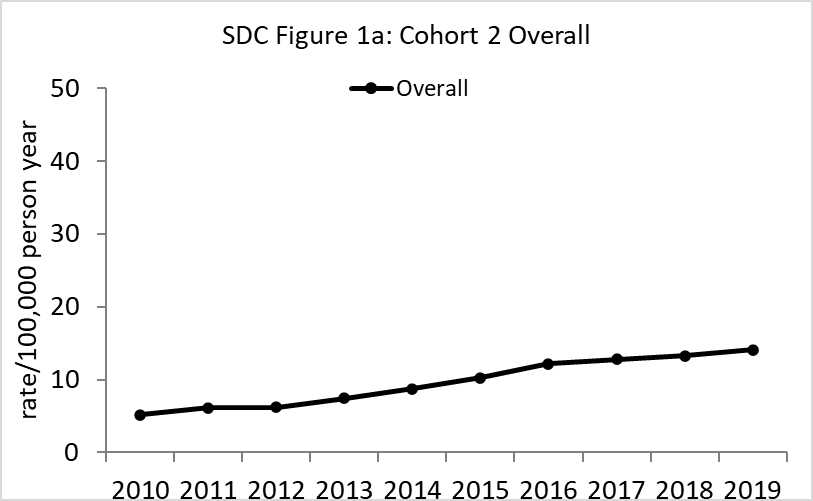 | 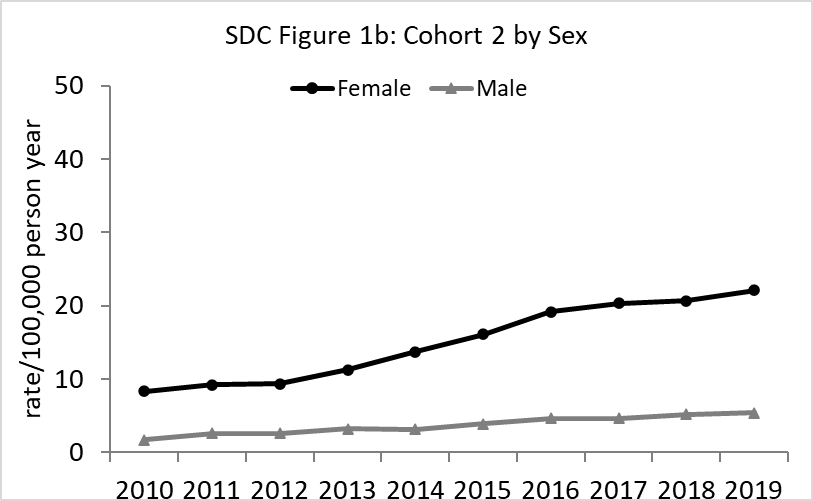 |
| --- | --- |
|  |  |
| 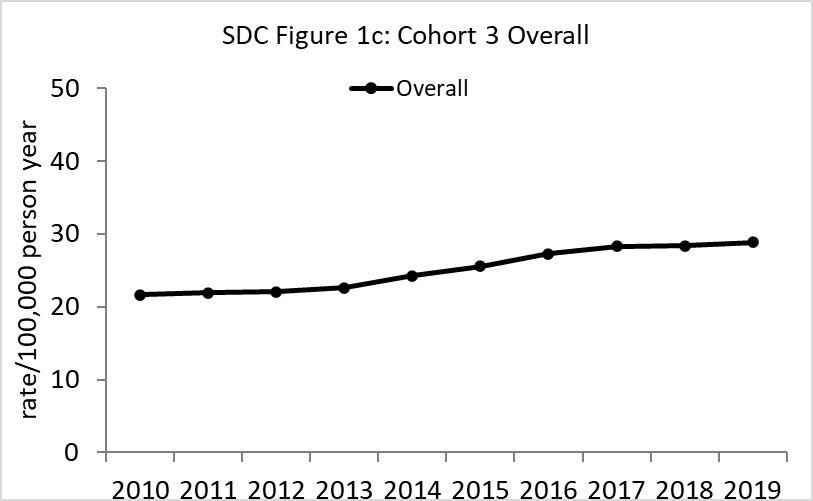 | 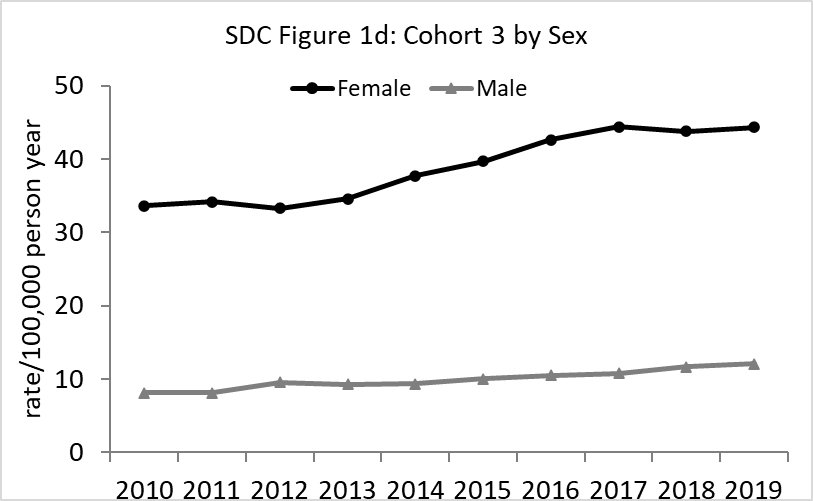 |
|  |  |
