# Supplementary material for: The prevalence of autoimmune hepatitis is rising: Estimates and trends from a large, multi-ethnic cohort in the United States
Source: Hepatol Commun. 2025 Oct 7;9(11):e0824. doi: 10.1097/HC9.0000000000000824 (PMC12506987; doi:10.1097/HC9.0000000000000824)
Supplement: Supplementary file 2 [file hc9-9-e0824-s002.docx]

| 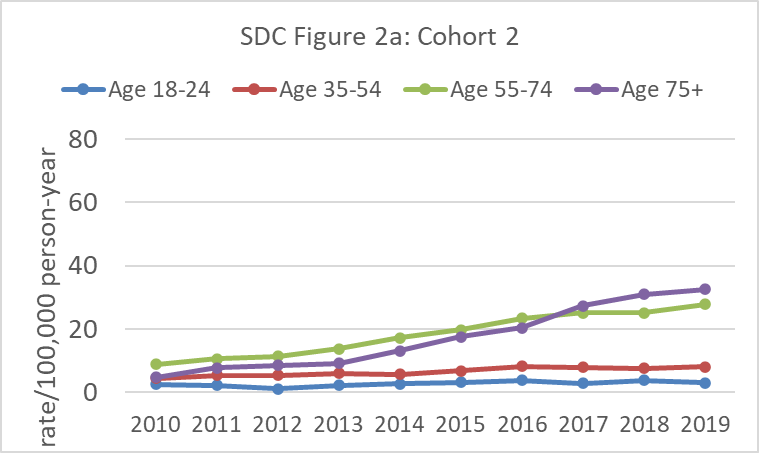 | 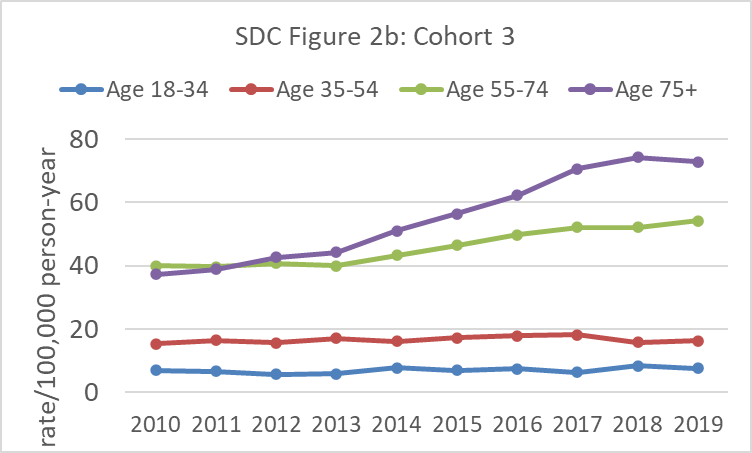 |
| --- | --- |
|  |  |
| 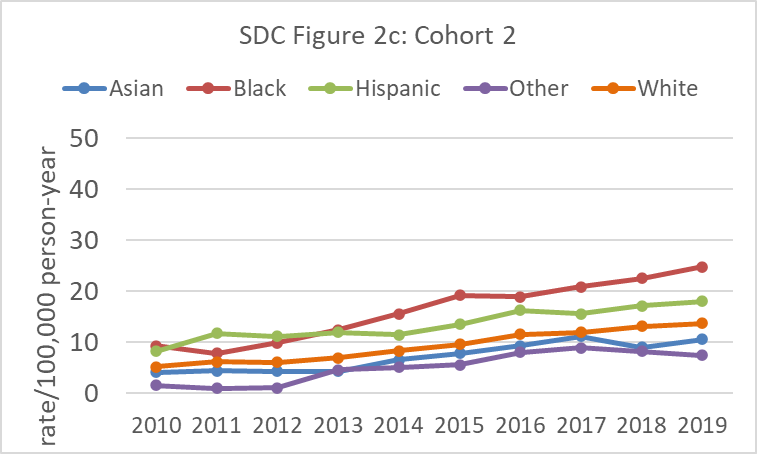 | 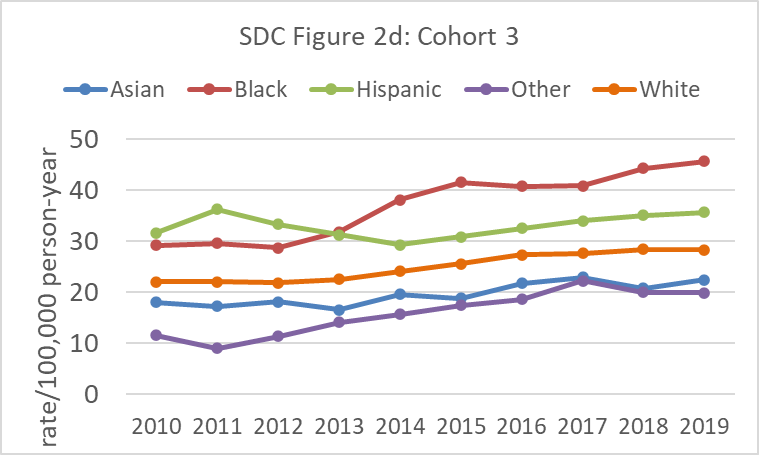 |

**Article Title:** Prevalence Estimates and Trends in Autoimmune Hepatitis in a Large, Multi-Ethnic Cohort in the United States

**First Author:** Jimmy Yao, MD

Supplemental Figure 2a-d, annual AIH prevalence per 100,000 individuals: a. Prevalence of AIH by age group for Cohort 2 (AIH confirmed by liver biopsy) among older adults rates rose from 4.8 to 32.5 from 2010-2019; b. Prevalence by age group for Cohort 3 (AIH by ICD code) among older adults rose from 37.2 to 72.8 from 2010-2019; c. Prevalence by race/ethnicity for Cohort 2 rose from 9.3 to 24.8 (Black), 8.3 to 18.0 (Hispanic), 5.2 to 13.8 (White) and 4.1 to10.6 (Asian) from 2010-2019; d. Prevalence by race/ethnicity for Cohort 3 rose from 29.2 to 45.6 (Black), 31.6 to 35.6 (Hispanic), 22.0 to 28.3 (White) and 18.0 to 22.5 (Asian) from 2010-2019.
