# Supplementary material for: The prevalence of autoimmune hepatitis is rising: Estimates and trends from a large, multi-ethnic cohort in the United States
Source: Hepatol Commun. 2025 Oct 7;9(11):e0824. doi: 10.1097/HC9.0000000000000824 (PMC12506987; doi:10.1097/HC9.0000000000000824)
Supplement: Supplementary file 3 [file hc9-9-e0824-s003.docx]

**Article Title:** Prevalence Estimates and Trends in Autoimmune Hepatitis in a Large, Multi-Ethnic Cohort in the United States

**First Author:** Jimmy Yao, MD

**Supplemental Table 1:** At Least Simplified Autoimmune Hepatitis Score Distribution

| “At Least” Simplified AIH***** Score | Cohort I  N (%) | Cohort II  N (%) |
| --- | --- | --- |
| 2 | 1 (0) | 0 (0) |
| 3 | 139 (12) | 11(1) |
| 4-5 | 457 (41) | 240 (32) |
| 6 | 272 (24) | 244 (33) |
| 7-8 | 260 (23) | 260 (34) |

*Autoimmune Hepatitis
